# Exploring the role of microbial biofilm for industrial effluents treatment

**DOI:** 10.1080/21655979.2022.2044250

**Published:** 2022-02-28

**Authors:** Indranil Chattopadhyay, Rajesh Banu J, T. M. Mohamed Usman, Sunita Varjani

**Affiliations:** a Department of Life Sciences, Central University of Tamil Nadu, Thiruvarur, India; b Department of Civil Engineering, PET Engineering College, Vallioor, Tirunelveli, India; c Paryavaran Bhavan, Gujarat Pollution Control Board, Gandhinagar, India

**Keywords:** Biofilm, quorum sensing, autoinducers, bioremediation, electro-active biofilms (EABs), genome editing

## Abstract

Biofilm formation on biotic or abiotic surfaces is caused by microbial cells of a single or heterogeneous species. Biofilm protects microbes from stressful environmental conditions, toxic action of chemicals, and antimicrobial substances. Quorum sensing (QS) is the generation of autoinducers (AIs) by bacteria in a biofilm to communicate with one other. QS is responsible for the growth of biofilm, synthesis of exopolysaccharides (EPS), and bioremediation of environmental pollutants. EPS is used for wastewater treatment due to its three-dimensional matrix which is composed of proteins, polysaccharides, humic-like substances, and nucleic acids. Autoinducers mediate significantly the degradation of environmental pollutants. Acyl-homoserine lactone (AHL) producing bacteria as well as quorum quenching enzyme or bacteria can effectively improve the performance of wastewater treatment. Biofilms-based reactors due to their economic and ecofriendly nature are used for the treatment of industrial wastewaters. Electrodes coated with electro-active biofilm (EAB) which are obtained from sewage sludge, activated sludge, or industrial and domestic effluents are getting popularity in bioremediation. Microbial fuel cells are involved in wastewater treatment and production of energy from wastewater. Synthetic biological systems such as genome editing by CRISPR-Cas can be used for the advanced bioremediation process through modification of metabolic pathways in quorum sensing within microbial communities. This narrative review discusses the impacts of QS regulatory approaches on biofilm formation, extracellular polymeric substance synthesis, and role of microbial community in bioremediation of pollutants from industrial effluents.

## Graphical Abstract

**Figure:**
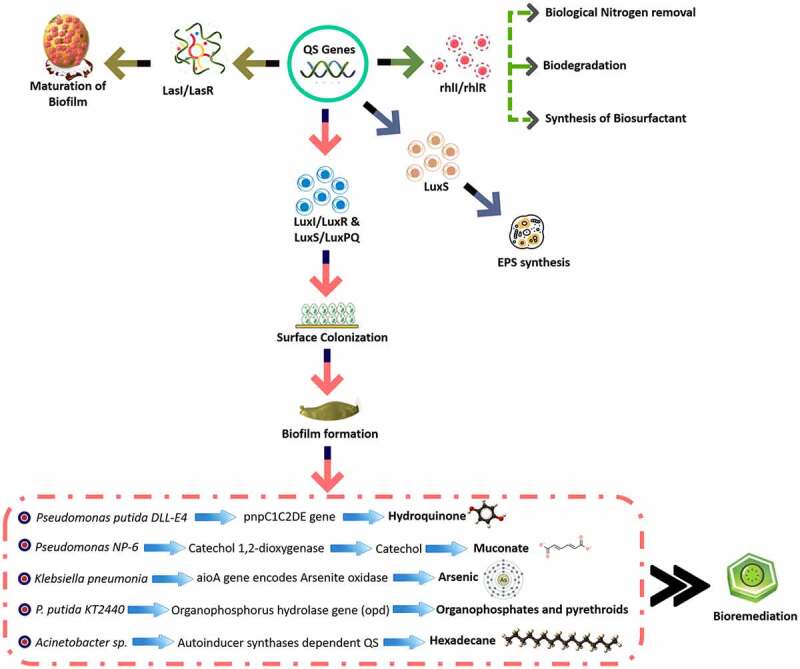

## 1. Introduction

Biofilm is an aggregation of single or multiple microbes that are adhered to biotic or abiotic surfaces irreversibly and are covered with a self-produced extracellular polymeric substance (EPS) [1]. The term ‘biofilm’ was introduced by Costerton et al. in 1978 [2]. The development of biofilm includes several steps such as adherence of microbiome to the surface, synthesis of EPS, the interaction between microbes through signaling molecules, and dissemination of microbial cells into planktonic form [3]. Biofilm protects microbes from the stressful environmental conditions, toxic action of chemicals, and antimicrobial substances [4]. The microbial cell population in biofilm ranges from 10^8^ to 10^11^ per g wet weight [5]. Biofilm formation by bacteria can occur in natural or anthropogenic environments. EPS of biofilm can hinder the action of pesticides, hydrocarbons, and heavy metals which are present in the close environment of biofilm [6]. Quorum sensing (QS) is defined as a communication procedure between bacterial cells in biofilm through chemical mediators which are known as autoinducers (AIs). The concentration of AI determines the expression of genes that control the population of a microbial cell [7]. QS contributes significantly to the evolution of biofilm and secretion of exopolysaccharides [8]. In gram-negative bacteria, the QS system is LuxI/LuxR type whereas QS in gram-positive bacteria is oligopeptide/two-component-type sensor histidine kinases [9]. Gram-negative bacteria use acylated homoserine lactone (AHL) as an auto-inducer whereas gram-positive bacteria use autoinducer peptide (AIP). Autoinducer-2 (AI-2) is used by both gram-negative and gram-positive bacteria in QS systems. Ligands such as AI-1, AI-2, AHL, and AIP-1 bind with their respective receptors such as LuxN, LuxP, LuxR, and AgrC to determine communication between bacterial species at the molecular level [10]. Different types of AIs from a variety of bacterial species are involved in environmental bioremediation such as remediation of toxic pollutants in soil and wastewater [11, 12]. In biological wastewater treatments, bacterial biofilm is predominated by gram-negative bacteria that use AHLs for communication. In activated sludges of wastewater treatment plants (WWTPs), AHLs-producing QS and AHLs-degrading QQ (quorum quenching) bacteria contribute significantly to controlling the development of biofilm in biological wastewater treatments [13]. Biofilm based environmental bioremediation is more eco-friendly and cost-effective as compared to other chemicals, physical, and thermal approaches. Microbial cells within biofilm provide resistance against the action of xenobiotic substances [14]. Biofilm is used for the treatment of wastewater where it is connected with biological oxygen demand (BOD), ammonia, nitrogen, nitrate, and dissolved O_2_. Nutrients of wastewater induce the growth of microbes and microbe derived metabolites which are used to remove the contaminants from the wastewater. Biofilm reactors such as biological contact oxidation tank, biological rotating disc, biological aerated filter, biofilm fluidized bed (BFB), moving bed biofilm reactor (MBBR) and integrated fixed-film activated sludge reactor are used to remove pollutants from domestic sewage and a variety of industrial wastewater. Biofilm reactors are capable to remove organic pollutants and nitrogenous substances at a high rate and they produce the least amount of sludge in wastewater treatment [15,16]. Microorganisms in biofilms on the surface of hydrocarbons are preferred for the removal of slowly degraded pollutants due to their high cell density, debilitate pollutants through biosorption, bioaccumulation, and biomineralization [17]. The utilization of biofilm in bioremediation depends on the interaction of microorganisms with xenobiotic substances in the environment. Immobilized cells in the biofilm are involved in the synthesis of cofactors and enzymes which are contributed significantly to bioremediation. Due to horizontal gene transfer (HGT) between the bacterial cells in biofilm, bacterial biofilms are more efficient for enhanced bioremediation as compared to planktonic cells [18].

Bibliometric analysis based on scientific search engines like PubMed revealed the applications of biofilm in bioremediation and wastewater treatments. The objective of this narrative review is to attempt to underline the current stage of characteristics of biofilm, the role of QS in biofilm development and their microbiological niches, and their role in bioremediation and wastewater treatment. The approaches for the characterization of biofilm using available analytical and molecular techniques are also briefly summarized. Role of exo-electrogens in biofilm growth in MFCs and biofilm associated EPS in bioremediation have been highlighted. Furthermore, this review also explores the need for genome editing technology in bioremediation.

## 2. Characteristics features of biofilm

**Figure 1.**
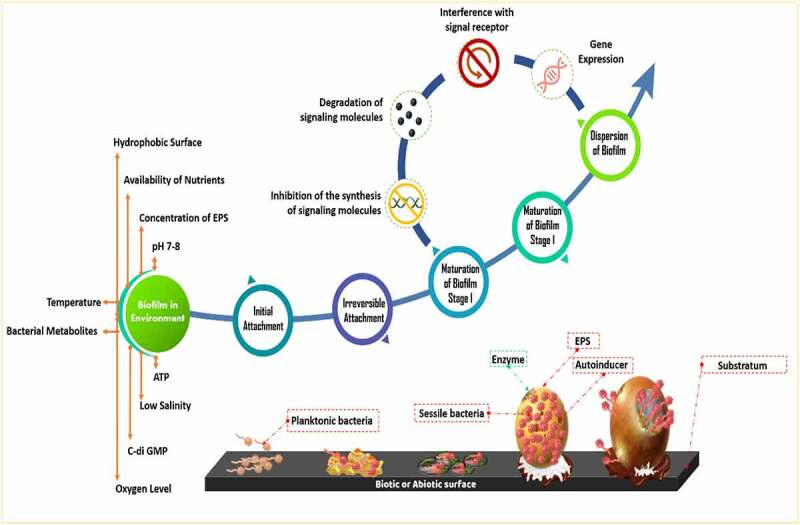
Stages of bacterial biofilm development along with factors involved in controlling biofilm formation.

**Figure 2.**
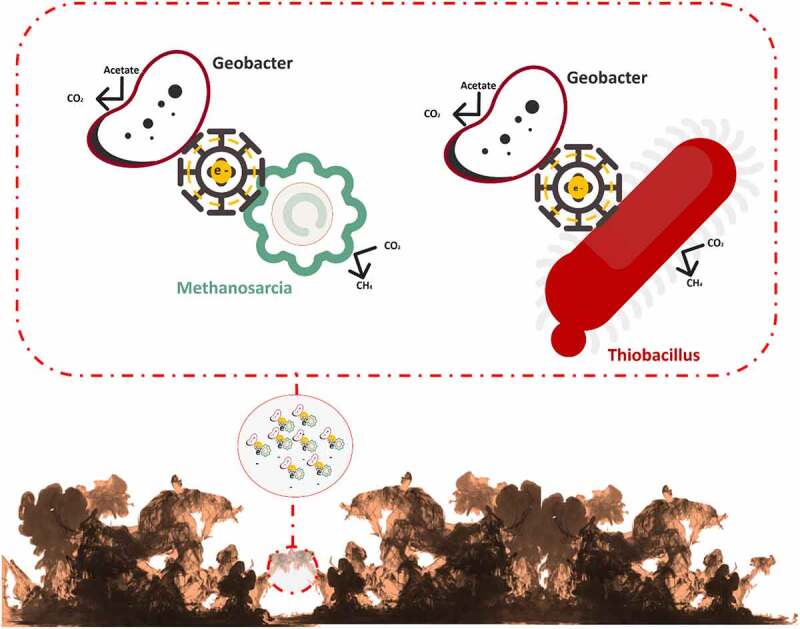
Syntrophic interaction in biofilms between fermentative bacteria and methanogenic archaea during anaerobic wastewater treatment.

In the natural environment, biofilms formation occurs between species belonging to algae, bacteria, fungi, and protozoa. The matrix of biofilm consists of either water or solvent. Free-living bacteria that do not adhere to surfaces are considered planktonic bacteria. The abundance of bacteria in planktonic cells becomes lower than bacteria in biofilms. Bacteria in biofilms can survive in adverse environmental conditions such as alterations of pH, presence of toxic substances and free radicals, and low amount of nutrient availability. The surfactants of EPS are used in solubilizing non-degradable compounds such as organic pollutants [19–21]. EPS matrix which is 0.2–1.0 mm thick shares 50% of total biofilm whereas microorganisms constitute the rest of the portion of biofilm [22]. Biosurfactants are involved in the manufacture of biofilm [23]. Water channels in a biofilm are involved in the diffusion of antimicrobial substances, oxygen, and nutrients [24]. Exopolysaccharide, cell population, pH, metabolites, oxygen level, and gene expression pattern are varied in different niches of biofilm [10]. The formation of biofilm is a dynamic process [25]. The evolution of biofilm has several steps such as reversible adherence of planktonic bacteria to the biotic or abiotic surface through Van der Waals interactions, irreversible adherence of bacteria through their flagella, fimbriae, and pilli, the proliferation of bacterial cells, secretion of EPS, QS, maturation of biofilm and dissemination of planktonic bacteria [26] (Figure 1). The water-binding capacity and mobility of the biofilm are responsible for the diffusion within the biofilm matrix which is composed of nutrients, polymers, and metabolites. D-glucuronic, D-galacturonic, and mannuronic acids contribute anionic property of biofilms. The presence of peptidoglycan, phospholipids, proteins, polysaccharides, DNA, and RNA is reported in biofilm [27]. Inorganic and organic substances which are secreted by the microbes provide a favorable condition for the adherence of microbial cells to the substratum [28]. Several saccharolytic enzymes are produced from microbial communities of biofilm. These enzymes are involved in the detachment of microbes from surfaces to colonize in a new location. Autoinducers are involved in the coordination of bacterial species in the biofilm [11]. HGT is responsible for the enhancement of genetic diversity such as genes responsible for the development of antibiotic and heavy-metal resistance and genes involved in the degeneration of environmental pollutants in a biofilm. Microbes in biofilms are communicated with each other through QS which regulates the expression of genes that drive biofilm formation [29]. Syntrophic interaction between bacteria is a well-documented phenomenon in biofilms. It occurs in between fermentative bacteria and methanogenic archaea during anaerobic wastewater treatment. Interspecies electron transfer (IET) is a crucial phase in syntrophic interactions. Syntrophic genera such as *Clostridium, Geobacter, Pelobacter*, and *Smithella* have highly abundant genes which are involved in the synthesis of diffusible signal factor (DSF) and c-di-GMP. Type IV pili are produced by c-di-GMP, which also regulates flagellar movement and biofilm matrix components that contribute to biofilm development and dissemination [30] (Figure 2). Heterotrophic bacteria such as *Alcaligenes, Bacillus, Escherichia coli, Nocardias, Pseudomonas, Streptococcus faecalis, Sphaerotilus, Thiobacillus*, and *Zoogleas* predominate in the biofilm [31].

### 2.1 Factors influencing morphology and behavior of biofilm

Environmental factors such as bacterial metabolites, oxygen level, pH, and nutrients determine the formation of biofilm [27]. Hydrophobic surface, low salinity, low temperature, and pH of 7–8 are favorable parameters for biofilm formation [16] (Figure 1). Dissolved oxygen content, temperature, and nutrients in wastewater are responsible to determine the bacterial abundance in the biofilm. The activity of biofilm is regulated by the level of adenosine triphosphate, dehydrogenase, deoxyribonucleic acid, and solid and volatile solid [16]. The activity of microbial communities in the biofilms is determined by the level of ATP. The aging of biofilm reduced the capability of biofilm in wastewater treatment [32]. pH alters the adhesion property of bacteria through alterations of its surface electrical charges. Repulsion occurs when the distance between the bacteria and the surface is 10–20 nm. EPS prevents microbes from the toxic effect of heavy metal and organic pollutants [33]. Biofilm formation is influenced by the concentration of EPS [34].

*Escherichia coli* K12 induced biofilm formation is regulated by aerobic granular sludge producing N-acyl-homoserine lactones (AHLs) [35]. The intracellular concentration of c-di-GMP is also involved in the formation of biofilm. A higher concentration of c-di-GMP enhances the adherence of bacteria to the surface and induces the formation of biofilm. Reduced concentration of intracellular c-di-GMP induces the dissemination of biofilm [36]. c-di-GMP regulated biofilm formation contributes significantly to wastewater treatment. Overexpression of c-di-GMP prevents biofilm formation caused by *Staphylococcus aureus*. Overexpression of the c-di-GMP regulated BalA protein promotes the spread of dissemination of *Pseudomonas aeruginosa* induced biofilm [37]. The transcription factor *RpoS* is involved in the formation and maturation of biofilm in *Escherichia coli* and *Pseudomonas aeruginosa* [38]. Overexpression of genes that encode adhesion and ribosomal proteins and downregulation of flagellar genes contribute significantly to biofilm formation [39].

### 2.2 Physiochemical properties of biofilm matrix

The biofilm matrix is also named EPS. EPS regulates the structure and stability of biofilm through the presence of Ca^+2^. The molecular weight of EPS is 500 to 2000 kDa. Exopolysaccharides contain sucrose-derived glucans, fructans, and cellulose. They are either polyanionic or polycationic. In *Pseudomonas aeruginosa*, exopolysaccharide such as alginate, Pel, and Psl37 are reported. Alginate contributes significantly in determining the formation of biofilm [40]. Amyloids are predominant components in the biofilms of wastewater treatment plants [41]. Extracellular DNA (eDNA) of the biofilm matrix provides the mechanical strength of biofilms and ensures the transfer of genetic information between bacterial cells [42]. The protein in EPS provides protection to biofilms against environmental stress [43].

## 3. Role of QS in biofilm development

QS depends on the population of microbial cells in the biofilm. QS also regulates the expression of genes that are involved in bioremediation, bioluminescence, production of antibiotics, and virulence factors [44]. AIs are produced by the bacteria as a signaling molecule during QS [45]. Boron-containing QS molecule (AI-2) is considered a common mode of the signal during communication. AI-2-mediated QS has been reported in *Deinococcus radiodurans, Escherichia coli, Salmonella typhimurium*, and *Vibrio cholerae*. The marine bacterium *Vibrio harveyi* uses *LuxLM* driven AI-1 (N-(3-hydroxybutanoyl)-homoserine lactone) for intraspecies communication and *LuxS* driven AI-2 (furanosyl borate diester) for interspecies communication [10]. In *Streptococcus mutans, comC* gene encodes competence-stimulating peptide (CSP) pheromone which induces biofilm formation [46]. Quorum sensing genes such as *lasI* and *rhlI* which encode AHL synthase contribute significantly to the growth of biofilm in *Pseudomonas aeruginosa* N6P6 which are involved in the degradation of polycyclic aromatic hydrocarbon (PAH) [47]. The AHL regulatory locus swr in *S. liquefaciens* and *S. marcescens* are involved in the manufacturer of biofilm [6]. In *P. aeruginosa, lasI/lasR* QS system is involved in the maturation of biofilm whereas *Rhl (rhlI/rhlR*) QS system is associated with the synthesis of biosurfactant [48].

### 3.1 QS in gram-negative bacteria

In Gram-negative bacteria, *LuxI* (autoinducer synthase) gene encodes acyl-homoserine lactone (AHL) autoinducer, and *LuxR* is the cytoplasmic AI receptor/DNA-binding transcriptional activator. The concentration of AHL enhances due to increased cell density. Due to its lipophilic nature, AHL can easily pass through the cell due to which intraspecies communication, biofilm formation, and EPS synthesis occur. The synthesis of AHL is species specific [49]. Acyl-homoserine lactone (AHL)-based quorum sensing has been reported in *Serratia liquefaciens* and *Rhodobacter sphaeroides* [27,50]. *Acinetobacter, Aeromonas, Burkholderia cepacian, Chitinimonas, Citrobacter, Enterobacter, Klebsiella, Pseudomonas aeruginosa, Pseudomonas putida*, and *Serratia liquefaciens* use AHLs for communication [51,52]. Homoserine lactone ring is a common feature of all AHLs [53]. Bacteria belong to Proteobacteria are commonly involved in the synthesis of AHLs. LuxI, LuxM, and HdtS protein families are involved in the synthesis of AHLs. The LuxI is mainly involved in the synthesis of AHL synthases. AHL-LuxR protein complex has been activated due to interaction between AHL signals and LuxR receptor and this complex binds to QS promoters to induce the transcription of genes that are regulated by the QS system. In *Pseudomonas aeruginosa*, AHL signals such as C4-HSL and 3-oxo-C12-HSL interact with RhlR and LasR for QS. Quorum quenching enzymes such as AHL-acylases, AHL-lactonases, and oxidoreductases are involved in the inhibition of AHL activity. *Pseudomonas strain* PAI-A and *Variovorax paradoxus* use AHL signals for their development. *Anabaena sp*. PCC7120, *Pseudomonas aeruginosa*, and *Ralstonia strains* are involved in the production of AHL-acylases. The presence of AHL biosensors has been reported in *Agrobacterium tumefaciens* NT1 plasmid pZLR4, *A. tumefaciens* A 136 (pCF218) (pCF372), *Chromoterbacium violaceum* CV026, and *E. coli plasmid* pSB401 [13,54]. *Acidobacteria sp., A. tumefaciens* C58, and *Bacillus sp*. strain 240B1 are involved in the synthesis of AHL-lactonases which are involved in the cleavage of the bond between homoserine lactone moiety and the acyl chain [55].

### 3.2 QS in gram‑positive bacteria

In gram-positive bacteria, AIP is used as an autoinducer which induces the structural diversity of biofilm. Membrane-associated ATP-binding cassette (ABC) transporter induces the secretion of AIP. The gene *agrD* encodes autoinducing peptides [56]. The Gram-positive bacterium *Exiguobacterium* sp is involved in inducing the AHL bioreporters, namely *Chromobacterium violaceum* CV026, *Agrobacterium tumefaceins* A136, and *E. coli* JM 109(psb1075) [57]. Gram-positive bacteria such as *Clostridium botulinum, C. perfringens, C. difficile, Enterococcus faecalis, Lactobacillus plantarum, Listeria monocytogenes*, and *S. aureus* are involved in the production of AIPs [18].

### 3.3 Mode of gene transfers in biofilm

Transformation, transduction, and conjugation are responsible for the transfer of genes among bacterial species in biofilms [27]. Horizontal transfers of plasmids carrying catabolic genes in biofilms modulate bacterial populations to enhance the degradation of xenobiotic compounds [58]. Genes responsible for the degradation of xenobiotic compounds such as PAHs are located in the bacterial plasmids or transposons. Goris et al. reported the effect of a transfer of plasmid pC1 of *Delftia acidovorans* tagged with a mini-Tn5 transposon encoding the gene for the oxidative deamination of 3-chloroaniline into *Pseudomonas putida* on activated sludge bacteria. Biofilm showed the highest frequency of gene transfer due to high microbial density [59]. Springael et al. reported transposon mediated 3-chlorobenzoate-degradative genes such as clc-element transfer from *P. putida* BN210 into other 3-chlorobenzoate mineralizing bacteria. HGT through conjugation and transformation is a common phenomenon in biofilm [60]. HGT of AHLs genes in *Novosphingobium* enhances the degradation of PAHs and pesticides [52]. Horizontal transfers of catabolic plasmids in biofilms result in a changed microbial population capable of degrading a variety of pollutants. Plasmids in the biofilm are immobile due to the quiescent nature of microbial cells in the biofilm. QS-mediated transformation, conjugative transfer, and prophage induction mechanisms have been reported in *B. subtilis, Rhizobium leguminosarum*, and *Rhodobacter capsulatus*. QS-mediated transformation enhances the catabolic potential of bacterial strains. Novosphingobium has aromatic ring hydroxylating dioxygenases and luxR homologous. Multiple mobile genetic elements which are present upstream of the luxR homologous region enhanced the HGT of AHLs genes [18].

## 4. Electro-active biofilm formation

Electrically potent microorganisms are involved in the formation of Electrochemically active biofilms (EABs) in wastewaters. They are involved in the electrochemical reactions in bioremediation. EABs are employed in bioelectrochemical systems (BESs) such as microbial fuel cells (MFC) and microbial electrolysis cells (MEC). Electrons are transported via pili/nanowires of some EABs. Bio-electrochemical treatment systems (BET) are used for wastewater treatment [61–63]. Metal-reducing bacteria use iron or manganese oxides as electron acceptors for the respiratory mechanism [64]. The development of biofilm in MFCs is determined by the bacterial species, temperature, pH, the pattern of substrate, and composition of electrode. Mixed culture biofilm generates more energy and shows higher potential for electron transfer as compared to pure culture biofilm [65]. The pH factor is involved in the redox potential in MFCs. At neutral pH, microbial enzymes are performed well for biofilm formation [66]. In the anaerobic anode compartment of MFCs, biofilm performs as a biocatalyst to synthesize protons and electrons through hydrolysis of the substrate such as ferricyanide, humic acid, thionin, and methylene blue [67]. In MFCs, microorganisms are involved in the formation of electricity through the transfer of electrons from bacterial cells toward the anode surface [68]. Exo-electrogens are involved in the formation of electro-active biofilms through the transmission of electrons through c-Cyts or pili. The exo-electrogenic potential of MFCs has been reported in β-Proteobacteria (*Rhodoferax*), γ-Proteobacteria (*Shewanella and Pseudomonas*), δ-Proteobacteria (*Aeromonas, Desulfuromonas, Desulfobulbus, Geobacter, and Geopsychrobacter*), Acidobacteria (*Geothrix*) and Firmicutes (*Clostridium*). C-type cytochromes (c-Cyts) and conductive pili (nanowires) which are located on the outer membrane-bound of *Geobacter* and *Shewanella* species involved in electron transmission. Type IV pili, c-Cyts pili, omcZ, and pilA are involved in the biofilm formation in *Aeromonas spp., Geobacter spp*., and *G. sulfurreducens* [65,69] (Table 1). Bacteria belonging to phylum Proteobacteria, Acidobacteria, and Firmicutes are involved in the generation of electricity. *Aeromonas hydrophila, Clostridium butyricum, Enterococcus gallinarum, Geobacter spp., Pseudomonas aeruginosa, Rhodoferax ferrireducens, Rhodobacter sphaeroide, Shewanella spp*., and *Shewadella oneidensis* MR-1 are involved in the generation of electricity in MFCs. *Clostridium beijerinckii, Clostridium butyricum*, and *Escherichia coli* K12 contributed production of hydrogen electrochemically on the anode [65] [70].,reported that the anodic bacterial community in MFCs fed domestic wastewater was overrepresented by *Geobacter metallireducens, G. sulfurreducens, G. lovleyi*, and *G. uraniireducens*. Proteobacteria, Firmicutes, and Bacteroidetes were highly abundant on the anode surface [71]. Spirochaetaceae (*Spirochaeta* spp.) and Methanobacteriaceae showed higher abundance in acetate accustomed biofilm whereas butyrate accustomed biofilm showed a higher abundance of bacterial communities such as Syngistaceae, Geobacteraeceae and Syntrophomonadaceae and Archeal communities such as Methanosarcinaceae [72]. Gram-positive bacteria showed a significant contribution to the EAB formation. *Thermincola* spp. of thermophilic MFCs is involved in the transmission of electrons through c-type cytochromes of its cell envelope. Thick layer anodic biofilms generate more electrical energy as compared to thin layer anodic biofilm [73].

**Table 1.** Bacteria involved in electrochemically active biofilms [EABs) formation in wastewater

Bacterial Taxa	Bacterial Species	Mode of electron transfer	Reference
δ-proteobacteria	*Geobacter sulfurreducens*	Type IV pili c-Cyts pili, omcZ, and pilA	[65,69]
	*Geobacter metallireducens*	c-Type cytochromes	
	*Desulfovibrio desulfuricans*	c-Type cytochromes	
γ-proteobacteria	*Shewanella oneidensis*	cytochrome MtrF heme network	
	*Shewanella putrefaciens*	c-Type cytochromes	
	*Pseudomonas putida*	Cyclic diguanosine-5’-monophosphate (c-di-GMP]	
	*Pseudomonas fluorescens*	Cyclic diguanosine-5’-monophosphate [c-di-GMP)	
	*Pseudomonas aeruginosa*	c-Type cytochromes	
Acidobacteria	*Geothrix fermentans*	Pyocyanin	

## 5. Application of biofilm-associated extracellular polymeric substances (EPS) and biosurfactants in bioremediation

The EPS ensures the binding of a bacterial cell to the surface of xenobiotics substances for the degradation of xenobiotics substances [74–76] [77].,reported that a higher amount of calcium dependent EPS augments *P. mendocina* NR802 induced degradation of PAHs. EPSs are hydrophobic in nature due to the presence of surfactants and lipids. Biosurfactants such as glycolipids, lipopetides, ionic lipids, and neutral lipids which are nontoxic and biodegradable are used in the enhancement of bioremediation of organic and inorganic pollutants. Biosurfactants are also involved in the development of biofilm through nutrient transport in biofilms via water channels [66, 78]. Biosurfactants such as rhamnolipids are reported in the biofilm matrix of *P. aeruginosa. rhl QS* system is involved in the synthesis of rhamnolipids in this bacterium. Biosurfactants are used in the bioremediation of polycyclic aromatic hydrocarbons (PAHs) [79–82]. The architecture of EPS in bacterial genera such as *Aeromonas, Bacillus, Burkholderia, Pseudomonas*, and *Xanthomonas* are involved in bioremediation and wastewater treatment through QS [76]. Bacteria such as *Enterobacter cloacear, Gordonia alkanivorans and Halomonas eurihalina* synthesize EPS having emulsifiers such as polysaccharides, proteins, and lipids which are involved in the degradation of hydrocarbons [83]. In a biofilm, oxidoreductase enzymes (laccase, polyphenoloxidase, and catalase) and EPS hydrolyase are involved in pyrene breakdown [6]. EPS played significantly in the wastewater treatment and bioremediation in soil [84].

## 6. Techniques applied for analysis and characterization of biofilm communities

**Figure 3.**
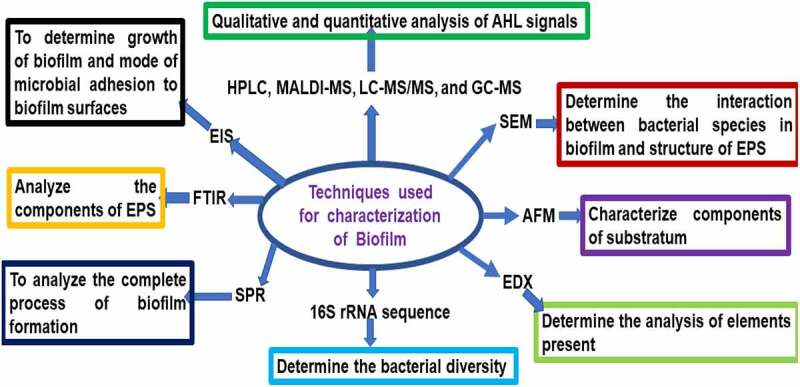
Biochemical and biophysical characterization of biofilm (16S rRNA sequence analysis can be applied to determine the bacterial diversity in the biofilm).

Scanning electron microscope (SEM), confocal laser scanning microscopy (CLSM), and atomic force microscopy (AFM) are used to determine the morphology of mature biofilms such as roughness, topography, and stiffness. AFM is used to characterize components of the substratum in the biofilm. SEM is also used to determine the interaction between bacterial species in biofilm and the structure of EPS. Energy Dispersive X-ray (EDX) spectroscopy is used to determine the analysis of elements present in biofilm. Fourier Transform Infrared (FTIR) spectroscopy is used to analyze the components of EPS in biofilm. Surface Plasmon Resonance (SPR) spectroscopy is used to analyze the complete process of biofilm formation. Electrical Impedance Spectroscopy (EIS) is used to determine the growth of biofilm and mode of microbial adhesion to biofilm surfaces. 16S rRNA sequence analysis is used to determine the bacterial diversity in the biofilm. Crystal violet assay is used to estimate the growth of biofilm [16]. High-performance liquid chromatography (HPLC), MALDI-MS, LC-MS/MS, and GC-MS have been used for qualitative and quantitative analysis of AHL signals [13] (Figure 3). 16S rRNA gene sequencing of the anodic microbial community in MFCs fed domestic wastewater determined the presence of *Geobacter metallireducens, G. sulfurreducens, G. lovleyi and G. uraniireducens* [65]. High performance liquid chromatography–mass spectrometry (HPLC–MS) analyses are used to determine AHL in activated sludge [85].

## 7. Role biofilms in bioremediation of organic compounds

Aerobic bacteria are involved in the biodegradation of polycyclic aromatic hydrocarbons (PAHs) through oxidation of the benzene ring by dioxygenase enzymes into dihydrodiols [86,87]. Several bacteria are involved in the degradation of naphthalene, anthracene, benzo (b) fluoranthene, dibenzo (a,h) and indeno (1,2,3-c,d) pyrene, and phenanthrene (Table 2). Biosurfactants such as surfactin, rhamnolipid, and sophorolipid are produced by B. subtilis, P. aeruginosa and Torulopsis bombicola which are involved in the bioremediation of PAH [21, 88, 89]. Glycolipids biosurfactants and polymeric biosurfactants are involved in the degradation of organic pollutants whereas biosurfactants of ionic lipids and lipopetides are involved in the insulation of pollutants [90–92]. *Sphingomonas sp*. augments the degradation of phenanthrene in the existence of biosurfactants [93]. *Chryseobacterium* sp., *Ochrobactrum intermedium, Klebsiella oxytoca, Pseudomonas alcaligenes, P. putida, Sphingobacterium* sp., and *S. maltophilia* are involved in the biodegradation of diesel in the presence of rhamnolipid [94]. Synthesis of biosurfactants may be augmented by the genetic modification of QS systems. Sulfate-reducing bacteria belong to *Desulfobacteriaceae* in a natural biofilm are used in the oxidation–reduction of sulfur [95]. Chlorinated aromatic compounds such as 2, 4-dichlorophenol (DCP) are present in the effluents of the chemical industry. Rotating perforated tube biofilm reactor which contains activated sludge culture enriched with *P*. putida containing microbial biomass is used to degrade DCP [96]. Microbial exopolymers in biofilms are used in the process of adsorption of chlorinated herbicide such as diclofop-methyl, and methyl 2-[4-(2,4-dichlorophenoxy) phenoxy] pyruvate [97]. Pseudoalteromonas haloplanktis TAC125 is involved in the bioremediation of aromatic compounds in marine environments into catechols [98]. Cycloclasticus zancles 78-ME, C. sp. DSM 27168, C. pugetii PS-1, C. sp. P1, and C. sp. PY97M are involved in the aerobic degradation of naphthalene, phenanthrene, and pyrene which are present in seawater and marine sediments [99]. Dehalococcoide is responsible for removing chlorinated ethenes [100]. Biofilms of Burkholderia sp. NK8 and P. aeruginosa PA01 are involved in the degradation of chlorinated benzoates [101]. Biofilms of Pseudomonas stutzeri T102 having naphthalene-degrading genes are involved in the bioremediation of naphthalene [102]. Pseudomonas sp. AKS2 is also involved in the degradation of low-density polyethylene (LDPE) through the formation of Biofilm on the surface of LDPE [103]. S. marcescens and S. liquefaciens are involved in the degradation of phenanthrene, diazinon, and catechol [104]. Burkholderia cepacian is also involved in the degradation of PAH [105]. QS regulates biofilm formation in *Pseudomonas aeruginosa* and *Pseudomonas putida*, both of which belong to the γ-proteobacteria. In *P. putida*, QS is controlled by *ppuI* and *ppuR*. Biofilm of *P. putida* is involved in the degradation of 3-chlorobenzoate due to the presence of 3-chlorobenzoate degradation cluster of plasmid within biofilms [60] (Table 2). EPS of *Enterobacter cloacear, Halomonas eurihalina*, and *Gordonia alkanivorans* are involved in the degradation of hydrocarbons [83].

**Table 2.** Bacteria involved in bioremediation of environmental pollutants

Environmental pollutants	Bacteria	Reference
Microplastics	*Acinetobacter calcoaceticus, Burkholderia cepacian, and Escherichia coli*	[115]
Poly[ε- caprolactone]	*Alcanivorax, Moritella, Pseudomonas, Psychrobacter, Shewanella, and Tenacibaculum*	[117]
Polyethylene	*Enterobacter asburiae* YT1 *and Bacillus* sp. YP1	[114]
Low-density polyethylene [LDPE]	*Pseudomonas* sp. AKS2	[103]
Plastics in seawater	*Arthrobacter, Aspergillus, Micrococcus, Pseudomonas, and Rhodococcus*	[113]
Benzoate, benzoylformate, mandelate, β-phenylpyruvate, and salicylate	*Azospirillum, Bradyrhizobium, Pseudomonas*, and *Rhizobium*	[110]
Ibuprofen	*Variovorax sp. Ibu-1 strain* *Sphingomonas sp. Ibu-2 strain*	[107,108]
Hydroquinone	*Azospirillum, Burkholderia, Brachymonas, Cupriavidus, Moraxella, Pseudomonas, Sphingomonas*, and *Variovorax*	[106]
Naphthalene	*Alcaligenes, Burkholderia, Bacillus firmus-* APIS272, *B. subtilis-*SBS526, *Mycobacterium, Polaromonas, Pseudomonas alcaligenes-* DAFS311, *Ralstonia, Rhodococcus, Sphingomonas, and Streptomyces* *Biofilms of Pseudomonas stutzeri* T102	[88,102]
Anthracene	*Bacillus firmus*-APIS272,*B. subtilis-SBS526, B. licheniformis, Burkholderia cepacia-*DAFS11, Beijerinckia sp., Mycobacterium sp., Nocardia sp., Pseudomonas alcaligenes-DAFS311, *Rhodococcus sp*. and *Sphingomonas sp.*	[88]
Phenanthrene	*Aeromonas, Acidovorax, Arthrobacter, Brevibacterium, Comamonas, Mycobacterium, and Sphingomonas*	[88]
Aromatic compounds in marine environments	*Pseudoalteromonas haloplanktis* TAC125	[98]
Naphthalene, phenanthrene, and pyrene of seawater and marine sediments	Cycloclasticus zancles 78-ME,*C. sp. DSM* 27168, *C. pugetii* PS-1,*C. sp*. P1, *and C. sp*. PY97M	[99]
Chlorinated benzoates	*Biofilms of Burkholderia* sp. NK8 *and P. aeruginosa* PA01 *Pseudomonas putida*	[60,101]
Phenanthrene, diazinon, and catechol	*S. marcescens, S. liquefaciens*	[104]
PAH	*Burkholderia cepacian, Alcanivorax, Arthrobacter, Aeromonas, Acinetobacter, Bacillus, Burkholderia, Cellulomonas, Corynebacterium, Dietzia, Enterobacter, Gordonia, Haemophilus, Mycobacterium, Microbulbifer, Micrococcus, Marinobacter, Pseudomonas, Paenibacillus, Rhodococcus, Sphingomonas*, and *Xanthomonas*	[105]

Biofilms manufactured by *Alcaligenes, Methylosinus Pseudomonas, Rhodococcus, and Sphingomonas* are involved in the degradation of organic compounds [27]. Bacterial strains such as *Azospirillum, Burkholderia, Brachymonas, Cupriavidus, Moraxella, Pseudomonas, Sphingomonas*, and *Variovorax* are involved in the biodegradation of hydroquinone. Nine genes of *Pseudomonas putida* DLL-E4 such as *pnpA, pnpR*, and *pnpC1C2DECX1X2* are involved in the metabolism of 4-nitrophenol. Gene pnpC which encodes hydroxyquinol 1, 2-dioxygenase is involved in the conversation of hydroxyquinol to maleylacetic acid. Gene such as pnpC1C2DE of *Pseudomonas putida* DLL-E4 is involved in the transformation of hydroquinone into metabolites of tricarboxylic acid cycle [106]. *Sphingomonas* sp. Ibu-2 strain of wastewater treatment plant is involved in the biodegradation of ibuprofen under aerobic conditions [107]. *Variovorax* sp. Ibu-1 strain of activated sludge is involved in the transformation of ibuprofen to trihydroxyibuprofen [108]. *Bacillus thuringiensis* B1 is also involved in the degradation of ibuprofen by using their ipfABDEF gene cluster in their genomes [109]. *Pseudomonas putida* F1 strain having cmt operon, *Micrococcus lysodeikticus* and *Bacillus* sp having homoprotocatechuate pathway, *Flavobacterium* strain having homogentisate degradation pathway, *Streptococcus rimosus* and *Pseudomonas cepacia* are involved in the degradation of ibuprofen. Gram-negative bacteria are involved in the aerobic degradation of phenylacetic acid by using (phenylacetyl)-coenzyme A ligase pathway. *Corynebacterium glutamicum* and *Pseudomonas* sp. KT2440 are involved in the degradation of benzoate by inducing the over expression of ABC transporter, cytochromes and NADH-dehydrogenases proteins [107]. Biofilm-forming soil bacteria such as *Azospirillum, Bradyrhizobium, Pseudomonas*, and *Rhizobium* are attracted to benzoate, benzoylformate, mandelate, β-phenylpyruvate, and salicylate through several QS genes such as rhlI [110] (Table 2). Understanding the interactions between microorganisms and chemicals is essential for successful bioremediation.

The biodegradation of plastics is involved in several steps such as bio-deterioration, bio-fragmentation, assimilation, and mineralization. In bio-deterioration, biofilm induces physical and chemical deterioration of plastic. In bio-fragmentation, enzymes such as oxygenases, lipases, and esterases which are secreted by the bacteria in biofilm are involved in the fragmentation of polymers in plastic into oligomers and monomers. In assimilation, oligomers that are generated due to bio-fragmentation are assembled in bacterial cells of biofilm as a carbon source. During mineralization, bacteria are involved in the oxidation of oligomers into CO_2_, N_2_, CH_4_, and H_2_O [111]. Wastewater treatment plants (WWTPs) are the main source of microplastics which are mostly present in sewage sludge [112]. *Arthrobacter, Aspergillus, Micrococcus, Pseudomonas, and Rhodococcus* are mainly responsible for the biodegradation of plastics in seawater, and plastic dumping sites [113] [114].,reported that *Enterobacter asburiae* YT1 and *Bacillus* sp. YP1 which are isolated from the gut of waxworms are involved in the degradation of polyethylene [115].,reported that *Acinetobacter calcoaceticus, Burkholderia cepacian*, and *Escherichia coli* are involved in the bioremediation of microplastics. Cyclobacteriaceae, Pirellulaceae, Phycisphaerales, and Roseococcus showed a higher abundance in biofilms on microplastics. Microbial enzymes are involved in the depolymerization of microplastics. Predominant microbial species of cold environments such as *Arthrobacter, Corynebacterium, Micrococcus, Pseudomonas, Rhodococcus*, and *Streptomyces* are also involved in biodegradation [116]. Bacterial genera such as *Alcanivorax, Moritella, Pseudomonas, Psychrobacter, Shewanella*, and *Tenacibaculum* from deep-sea sediment are involved in the biodegradation of poly (ε-caprolactone) [117]. Organic carbon which is derived from plastic of seawater induces the growth of heterotrophic microbes [118] (Table 2). Polyethylene terephthalate (PET) is commonly used in bottles and synthetic fibers. Due to its long carbon chains bearing aromatic rings, PET is more stable in the environment and it is difficult to biodegrade. *Pseudomonas mendocina, Ideonella sakaiensis, Nocardia* sp., and *Thermobifida fusca* are involved in the degermation of PET by using enzymes such as lipases and esterases [119]. *Rhodococcus ruber* is involved in the metabolism of polyethylene by using laccase enzyme [120]. Algal derivatives such as polyhydroxyalkanoates (PHAs) from cyanobacteria, and starch from microalgae are involved in the synthesis of bioplastic [121].

## 8. Role of quorum sensing in biological wastewater treatments

QS signals such as AHLs of Agrobacterium, Aeromonas, and Pseudomonas contribute significantly to wastewater treatment [11]. AHLs enhance the degradation of phenol in wastewater treatment [122]. QS signals of *Acinetobacter* sp. DR1 is involved in the degradation of hexadecane [123]. The rhl QS system and expression of catechol 2, 3-dioxygenase in *P. aeruginosa* is involved in the degradation of benzoate, phenanthrene, and phenol [124]. QS induces the development of aerobic granule (AG) through aggregation in wastewater treatment [11]. AHL producing bacterial genera such as *Aeromonas* and *Pseudomonas* showed a higher abundance in Activated sludge (AS) which are commonly used for the purification of domestic and industrial wastewater 6. The structure and metabolic activity of activated sludge in wastewater treatment are controlled by N-heptanoyl-L-homoserine lactone [125]. AHLs enhance the expression of amoA genes of ammonia-oxidizing archaea and bacteria in wastewater treatment [126]. Biofilms have both favorable roles such as granular sludge, and moving bed biofilm reactors, and the adverse role such as membrane biofouling in wastewater treatment [51]. AI-2 is involved in the development of biofilm by *Escherichia coli* through the synthesis of EPS [127]. AI-2 impacts aerobic granulation through the synthesis of EPS [128]. Quorum quenching (QQ) activity is reduced by the granulation [129]. AHL regulates the synthesis of EPS by *P. aeruginosa* through *LuxS* and *Esal/EsaR* systems [130]. Bacteria belonging to Flavobacterium and Xanthomonadaceae showed higher abundance in activated sludge with an upsurge of AHLs concentrations [51]. The abundance of bacteria belong to *Anaerolinea, Bacteroidetes, Proteiniphilum*, and *Syntrophobacter* become elevated in the presence of short-chain AHLs [131]. The *RhlI/RhlR* system of *P. aeruginosa* is involved in the treatment of industrial and municipal wastewater particularly in the degradation of phenol. *Acinetobacter sp*. is involved in the metabolism of hexadecane through autoinducer synthases dependent QS. AHL is used for the removal of pollutants from wastewater through the synthesis of biofilm and biosurfactant, horizontal gene transfer, and expression of genes that are involved in the xenobiotic metabolism. QQ enzymes such as lactonases (quorum-quenching N-acyl-homoserine lactonases), acylase amidases such as amidohydrolases or acylases, reductases, and cytochrome oxidases are involved in the degradation of AHLs. Management of microbiome at the community and molecular level through QS contributes significantly to the bioremediation of organic pollutants [51]. *Pseudomonas* sp. 1A1, *Variovorax paradoxus* VA1-C and *Rhodococcus* sp. BH4 showed inhibitory activity against QS signals in the presence gamma-caprolactone (GCL) of activated sludge [132] [129].,reported the presence of QS and QQ genes in activated sludge of wastewater treatment facilities by using metagenomic approaches. AHL-quenching bacteria were present in a higher proportion as compared to AHL-producing bacteria in aerobic granules [133]. Overgrowth of biofilm is responsible for the poor performance of membrane biofilm reactors (MBfR) and moving bed biofilm reactors (MBBR). QQ-bacteria such as *Acinetobacter* sp.,*Afipia* sp.,*Microbacterium* sp.,*Micrococcus* sp.,*Pseudomonas* sp., and *Rhodococcus* sp are responsible for the prevention of the growth of biofilm [134]. Quorum quenching activity was also exhibited by *Brevundimonas, Comamonas, Mesorhizobium, Pedobacter*, and *Variovorox* [135]. *Candida albicans* synthesize QQ agents such as farnesol which inhibits the growth of biofilm on MBR [136] (Table 3). *Geobacter metallireducens* is involved in the reduction of Mn (IV) to Mn (II) whereas *G. sulfurreducens and G. metallireducens* are involved in the reduction of U(VI) to U(IV) and Cr (VI) to less toxic Cr (III) [137]. Acetonitrile which is commonly discharged through industrial wastewater becomes hazardous to aquatic organisms. *B. subtilis* E2, E3, and N4 as well as *Rhodococcus rhodochrous* BX2, are used to degrade acetonitrile [138a]. *B. subtilis* N4-pHT01-nit is involved in the degradation of acetonitrile along with Moving bed biofilm reactor (MBBR) [139]. *Arthrobacter sp., Pseudochrobactrum saccharolyticum* LY10 sp., *Aspergillus carbonarius* and *P. mendocina* NR802 are involved in the elimination of xenobiotic substances and toxic heavy metals [140,141]. *P. aeruginosa* CGMCC1.860 is involved in the synthesis of AHL molecules such as C4HSL and C6HSL which are involved in the biodegradation of aromatic compounds [142]. Sulfate reducing bacteria (SRB) such as *Desulfovibrio* are involved in the bioremediation of wastewater containing Cu^+2^ [143]. Biodegradation of bisphenol A (BPA) from wastewater is aided by *Acidovorax* sp., *Luteimonas* sp., and *Pseudomonas* sp [144]. *Mezorhizobium, Devosia, Pseudoxanthomonas, Bosea*, and *Paracocci* have been found in wastewater treatment systems that use nitrogen metabolism [145]. QS and QQ have been found to influence the synthesis of EPS, biofilm formation, and biodegradation of organic pollutants and biofouling management in wastewater treatment.

**Table 3.** Quorum sensing (QS] and quorum quenching [QQ) bacteria involved in treatment of municipal or industrial wastewater

Bacteria	Mode of action of QS and QQ system involved in degradation of pollutants in wastewater	Reference
*Acinetobacter* sp. DR1	QS signals involve in the degradation of hexadecane	[123]
*Pseudomonas aeruginosa*	rhl QS system and expression of catechol 2, 3-dioxygenase involved in the degradation of benzoate, phenanthrene, and phenol	[124]
*Pseudomonas aeruginosa*	RhlI/RhlR system involved in the treatment of industrial and municipal wastewater particularly in the degradation of phenol	[51]
*Pseudomonas* sp. 1A1, *Variovorax paradoxus* VA1-C *and Rhodococcus* sp. BH4	Showed inhibitory activity against QS signals in the presence gamma-caprolactone [GCL] of activated sludge	[132]
*Pseudomonas aeruginosa* N6P6	Quorum sensing genes such as lasI and rhlI which encode AHL synthase involved in degradation of polycyclic aromatic hydrocarbon [PAH]	[47]
*Streptococcus mutans*	Competence-stimulating peptide [CSP] pheromone involved in Biofilm formation	[46]

## 9. Application of moving bed biofilm reactor (MBBR) in waste water treatment plant (WWTP)

**Figure 4.**
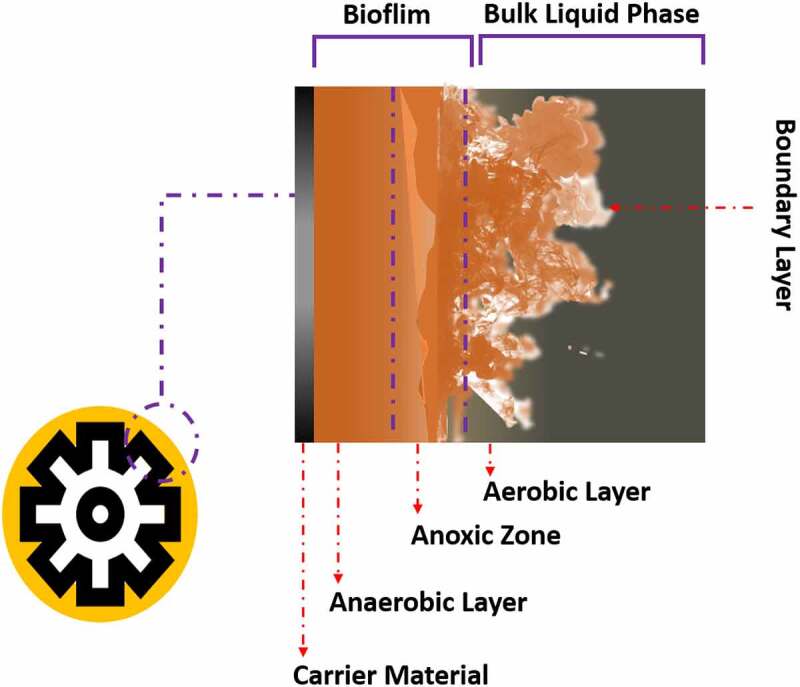
Schematics of moving bed biofilm reactor (MBBR) used for wastewater treatment.

Nutrients of wastewater are a major source of contamination in the environment [146–148]. Biological Nutrient Removal (BNR) is not efficient to remove the nutrients from wastewater due to the presence of microorganisms [149]. MBBR has been considered an efficient system for introducing biomass as biofilm [150,151]. Moving bed biofilm technology has several advantages such as reduced obstruction, resistance against pH, temperature, and toxic substances, and maintains of biomass as a biofilm on carriers [152,153]. The carrier materials such as polyethylene, polypropylene, and polyvinyl chloride are responsible for the construction of biofilm and microbial diversity in the biofilm. The amino functional group (-NH_2_) into polyethylene and polypropylene increases the thickness and density of biofilm [154] (Figure 4). Membrane bioreactors (MBRs) are considered the best wastewater treatment approach. Membrane biofouling is one of the most serious threats in MBR applications. In membrane biofouling, unwanted microorganisms become assemble and form biofilm on the membrane surface through attachment and expansion [155]. Bacteria such as *Aeromonas, Citrobacter, Enterobacter, Klebsiella, Pseudomonas, and Serratia* are involved in the growth of biofilm on the membrane surface through AHL-based QS system [156, 157]. Enzymes such as acylases or lactonases which are involved in the quenching of AHL activity showed a significant role in controlling the membrane biofouling in MBRs. Bacteria such as *Anabaena* sp. PCC7120, *A. tumefaciens* C58, *Bacillus sp. strain* 240B1, *P. aeruginosa* PAO1, *Rhodococcus erythropolis* strain W2, and *Ralstonia* sp. XJ12B is responsible for the synthesis of produce quorum quenching enzymes [156]. QQ technology is an appealing and cost-effective method of biofouling control in MBRs.

Biofilms based WWT technologies are used to remove contaminants such as organic and nitrogenous substances from wastewater. In the sludge bed of the bioreactor, the microbiota adheres to the sludge granules. Anaerobic bioreactors are involved in the generation of methane as a byproduct through the metabolism of organic substances [158]. Prokaryotes belong to Phylum Euryarchaeota and six orders such as *Methanobacteriales, Methanococcales, Methanomicrobiales, Methanosarcinales, Methanopyrales*, and *Methanocellales* are involved in methane synthesis [159]. Methanobacterium, Methanosaeta, and Methanosarcina showed higher abundance in the fixed-bed reactor and seven fluidized-bed reactors that are involved in the treatment of industrial wastewaters. Methanobacteriales and Methanomicrobiales showed co-existence in fixed-film anaerobic reactors [160]. *Methanobacteriales, Methanomicrobiales*, and *Methanospirillum* showed abundance in fixed-bed anaerobic baffled reactors (FABRs) [161] [162].,reported that *Methanomethylovorans hollandica, Methanobacterium aarhusense, Methanobacterium subterraneum* and *Methanolinea tarda* were highly abundant in the granular bioreactor. Methanomicrobiales showed higher abundance in anaerobic fixed-bed biofilm systems at low temperatures [163]. Methanobacteriaceae, Methanomicrobiaceae, and Methanosarcinaceae showed higher abundance in the packed-bed biofilm reactors [164]. Methanogenic archaea play a critical role in anaerobic wastewater treatment.

Genome editing tools such as Clustered regularly interspaced short palindromic repeats (CRISPR-Cas), Transcription-activators like effector nucleases (TALEN), and zinc finger nucleases (ZFNs) are presently involved in the enhancement of bioremediation. Among them, the CRISPR-Cas system which consists of guide RNA (gRNA) linked crisper derived RNA (crRNA) and transacting antisense RNA (trcRNA) is widely used in *Pseudomonas* and *Escherichia coli*. The CRISPR system is also used to express genes involved for bioremediation in *Achromobacter sp. HZ01, Comamonas testosterone*, and *Rhodococcus ruber* TH. Genome editing enhances the survival of bacteria in a toxic environment. Metabolic engineering is involved in the modification of microbial enzymes such as esterases, monooxygenases, oxidases, oxidoreductases, and phenoloxidases which are involved in bioremediation. Enzyme-based bioremediation is compatible with our ecological condition [165]. In *Klebsiella pneumonia, aioA* gene encodes arsenite oxidase which is involved in the bioremediation of arsenic [166]. Catechol 1,2-dioxygenase of *Pseudomonas* NP-6 is involved in the degradation of catechol into muconate compounds [167]. Insecticides used in agricultural fields such as organophosphates (OP) and organochlorines (OC) entered into the water. Genetically modified *P. putida* KT2440 has been used in the bioremediation of organophosphates and pyrethroids [168]. Enzymes (haloalkane dehalogenase, wild-type haloalcohol dehalogenase, and wild-type epoxide hydrolase) of genetically modified *E. coli* strain have been utilized in the degradation of 1, 2, 3-trichloropropane [169].

Insertion of organophosphorus hydrolase gene (*opd*) and *pnp* operon encoding enzymes in *P. putida* is involved in the bioremediation of organophosphorus and paraoxon [170]. In *Rhodococcus opacus R7, pobA* and *chcpca* gene clusters are involved in the bioremediation of naphthenic acid. Expression of the aliA1 gene which encodes fatty acid CoA ligase is involved in the degradation of alicyclic naphthenic acid [171]. The enzyme oxygenase is involved in the degradation of organic compounds. OxDBase provides information about the enzyme oxygenases which are involved in bioremediation [172–174]. Bionemo (Biodegradation Network Molecular Biology) database provides information about the expression of genes involved in biodegradation [175]. EAWAGBBD PPS database provides information about the pathways involved in the biodegradation of 1-naphthyl-N-methyl carbamate [176]. *Alcaligenes xylosoxidans* and *Pseudomonas stutzeri* showed enhancement of biodegradation of polychlorinated biphenyls in the presence of biphenyl [177]. Squamocin induces biofilm formation in *Bacillus atrophaeus* CN4 which is involved in the bioremediation of naphthalene [178]. *Cyanobacterium, Phormidium autumnale* UTEX1580 has been involved in the metabolism of indigo dye which is released from textile industries [179]. Cloning of flavin-diffusible monooxygenase encoding genes such as *cphC-I* and *cphB* of *Arthrobacter chlorophenolicus* in *E. coli* enhances bioremediation of 4-chlorophenol [180]. The application of synthetic biology approaches in microbes improves the effectiveness of microbial bioremediation processes for specific contamination [181,182,183].

## 10. Future prospects and conclusions

Aggregation of toxic xenobiotic substances in the environment is increasing, therefore it is essential to reduce concentration of xenobiotic substances through different approaches. Microbes based removal of environmental pollutants is ecofriendly. Biofilm based bioremediation approach is one of the potential approaches for reducing the level of pollutants in the environment. Biofilms are natural habitats where bacterial cells exchange genetic material, signaling molecules, and metabolites. QS is responsible for the interaction between bacterial cells of different species. To understand bioremediation of environmental contaminated sites such as wastewaters, it is essential to understand bacterial diversity in biofilm by using metagenomic approaches such as 16s rRNA sequencing. Microbiome based bioremediation process has now emerged as an essential approach in the global scenario. The development of genetically engineered biofilm formation enhances bioremediation of pollutants. Synthetic biology approaches are made to increase the efficiency of microbial enzymes in bioremediation. QS technology enhances bioremediation process in industrial wastewater treatment plants. QS regulates the development of biofilm, synthesis of EPS, and biosurfactant which contribute significantly to the removal of heavy metals and organic substances from the ecosystem. In WWTPs, QS and QQ are responsible for aggregation, colonization, granulation, removal of nutrients, metabolism of organic substances, and biofouling. It is essential to understand the role of AHL-based QS and QQ systems in wastewater treatments. Genome editing, omic based approaches, and metabolic engineering along with in silico databases and bioinformatics tools may open a new avenue for biofilm based bioremediation research.
